# Prognostic necroptosis-related gene signature aids immunotherapy in lung adenocarcinoma

**DOI:** 10.3389/fgene.2022.1027741

**Published:** 2022-11-25

**Authors:** Yuqi Song, Jinming Zhang, Linan Fang, Wei Liu

**Affiliations:** ^1^ Department of Thoracic Surgery, First Hospital of Jilin University, Changchun, China; ^2^ First Hospital of Jilin University, Changchun, China

**Keywords:** lung adenocarcinoma, necroptosis, gene signature, prognosis, immunotherapy, chemotherapy

## Abstract

**Background:** Necroptosis is a phenomenon of cellular necrosis resulting from cell membrane rupture by the corresponding activation of Receptor Interacting Protein Kinase 3 (RIPK3) and Mixed Lineage Kinase domain-Like protein (MLKL) under programmed regulation. It is reported that necroptosis is closely related to the development of tumors, but the prognostic role and biological function of necroptosis in lung adenocarcinoma (LUAD), the most important cause of cancer-related deaths, is still obscure.

**Methods:** In this study, we constructed a prognostic Necroptosis-related gene signature based on the RNA transcription data of LUAD patients from The Cancer Genome Atlas (TCGA) and Gene Expression Omnibus (GEO) databases as well as the corresponding clinical information. Kaplan-Meier analysis, receiver operating characteristic (ROC), and Cox regression were made to validate and evaluate the model. We analyzed the immune landscape in LUAD and the relationship between the signature and immunotherapy regimens.

**Results:** Five genes (RIPK3, MLKL, TLR2, TNFRSF1A, and ALDH2) were used to construct the prognostic signature, and patients were divided into high and low-risk groups in line with the risk score. Cox regression showed that risk score was an independent prognostic factor. Nomogram was created for predicting the survival rate of LUAD patients. Patients in high and low-risk groups have different tumor purity, tumor immunogenicity, and different sensitivity to common antitumor drugs.

**Conclusion:** Our results highlight the association of necroptosis with LUAD and its potential use in guiding immunotherapy.

## Introduction

As the most important cause of cancer death, lung cancer has been a major research topic for clinicians and researchers (Sung et al., 2021). Non-small cell lung cancer (NSCLC), the most important type of lung cancer, accounts for 85% of the total incidence of the disease (Chen et al., 2014). Slow-growing, insidious-developing lung adenocarcinoma (LUAD) is the most common pathological type of NSCLC. It is prone to hematogenous metastasis, so some patients are often diagnosed at a late stage, which deprives them of the opportunity for surgery and their clinical prognosis is poor (Devarakonda et al., 2015). The advent of targeted therapies and immunotherapy has brought better options for such patients, but most of them have no mutation in the driver gene or do not respond to a single immune checkpoint inhibitor (ICI) (Matter et al., 2020; Santarpia et al., 2020). In recent years, with the development of RNA sequencing, microarrays, and other “Omics” technologies, a series of new potential markers driving tumor cell formation have been identified and progressively applied in the clinic. The average 5-year survival rate of LUAD patients, although significantly improved, is still less than optimal.

Necroptosis is a phenomenon of cellular necrosis resulting from cell membrane rupture by the corresponding activation of Receptor Interacting Protein Kinase 3 (RIPK3) and Mixed Lineage Kinase domain-Like protein (MLKL) under programmed regulation (Degterev et al., 2005). It has a proper regulatory mechanism. With the advancement of basic research, necroptosis has been found to be not only involved in the inflammatory pathological mechanism of the body (Khoury et al., 2020) but also closely related to the development of tumors and drug resistance. Preliminary studies suggest that necroptosis has a “double-edged sword” role in tumor pathology, which can exert either tumor-suppressive or tumor-promoting effects (Raposo et al., 2015; Liu et al., 2016; Hänggi et al., 2017). On the one hand, inducing necroptosis can remove chemotherapy-resistant tumor cells; on the other hand, it may also kill normal cells and lead to inflammatory responses that promote tumor progression and metastasis (Gong et al., 2019).

Immunotherapies, represented by ICIs such as various antibodies against cytotoxic T lymphocyte-associated antigen 4 (CTLA-4), programmed cell death 1 (PD-1), and programmed cell death ligand 1 (PD-L1), are designed to stimulate the patient’s immune system to trigger an effective anti-tumor immune response (Rosenberg, 2014). Despite its emerging and encouraging results, increased immune tolerance is frequently documented in many cancer types (Bonavida and Chouaib, 2017). Furthermore, a large proportion of studies have also highlighted the potentially enormous impact of necroptosis-driven immunogenic features in tumor immunology, for example, the induction of necroptosis can act synergistically with ICIs to enhance their antitumor activity in drug-resistant tumors (Tang et al., 2020). These close and complex relationships suggest that necroptosis may be an important target for tumor progression and may provide new strategies for tumor immunotherapy and prognosis (Philipp et al., 2016). However, to date, the mechanism of the role of necroptosis in LUAD is unclear, and its relationship with immunotherapy and prognosis has been little studied.

The aim of this study was to construct a robust prognostic model of Necroptosis-Related Genes (NRGs) by bioinformatics algorithms to predict the survival probability of LUAD patients at different periods. We will also explore the functional pathways and signaling pathways involved in key genes and their relationship with immune cell infiltration, tumor mutation burden, immunotherapy, and drug sensitivity, to assist in individualized and precise treatment.

## Materials and methods

### Data acquisition

RNA transcriptome information and clinical information of LUAD patients were obtained from The Cancer Genome Atlas (TCGA) database and the GSE72094, GSE50081 datasets in Gene Expression Omnibus (GEO) database, respectively. The RNA-seq transcriptome data were converted to transcript volume per million (TPM) values, and the R “limma” and “sav” packages were applied for batch correction and normalization of RNA-seq from both platforms. After excluding samples with incomplete clinical information or gene expression data, 504 patients from TCGA and 398 patients from the GSE72094 dataset with LUAD were included in the downstream analysis, and 127 patients from the GSE50081 dataset were used for external validation. 17 NRGs (RIPK1, RIPK3, MLKL, TLR2, TLR3, TLR4, TNFRSF1A, PGAM5, ZBP1, NR2C2, HMGB1, CXCL1, USP22, TRAF2, ALDH2, EZH2, NDRG2) were obtained from literature reviews of previous related studies (Petersen et al., 2015; Choi et al., 2019; Lou et al., 2019; Malireddi et al., 2019; Wen et al., 2020; Xia et al., 2020; Zhu et al., 2020; Cheng et al., 2021; Roedig et al., 2021). The relative position of these genes to the chromosomes was visualized using the R “RCircos” package. The Human Protein Atlas database (https://www.proteinatlas.org/) was used to display the expression of proteins encoded by NRGs.

### Construction and validation of a necroptosis-related prognostic signature

Sample data from the TCGA and GSE72094 dataset was combined, including expression data of NRGs and patients’ survival data. A univariate Cox regression analysis was performed to obtain genes significantly associated with prognosis. After that, we randomly divided the patients into Train and Test sets (632 in the Train set and 270 in the Test set). The R “glmnet” package was used to perform LASSO regression analysis on the prognostic data and to optimize the penalty function using cross-validation. A prognostic signature consisting of genes related to necroptosis was developed to predict the prognosis of LUAD patients. The formulae are as follows:

Risk Score=coefficients∗expressing values of A gene+coefficients∗expressing values of B gene+…

Using the “CatPredi” software package, an R package allows the user to categorize a continuous predictor variable in a logistic or a Cox proportional hazards regression setting by maximizing the discriminative ability of the model, we determined the optimal two cut-off values for the Train and Test sets separately, splitting each set into a low-risk group and a high-risk group. Kaplan-Meier analysis was used to plot the overall survival (OS) curves for each set. In this study, OS was defined as the duration from the date of diagnosis to death or last follow-up, with no restriction on the cause of death. The R “timeROC” package was used to generate subject operating characteristic (ROC) curves, and the area under the curve (AUC) of the ROC curves was measured to show the sensitivity and specificity of the model.

In addition, we performed univariate and multivariate Cox regression analyses of the validity of the risk score as an independent prognostic indicator. The clinical characteristics of high and low-risk patients were compared using the R “pheatmap” package to explore the correlation between risk scores and clinicopathological variables.

### Nomogram construction and verification

We constructed a nomogram based on 902 samples from all TCGA + GSE72094 datasets to predict the survival rate of patients at 1, 2, and 3 years using pathological staging and risk score information. We then plotted ROC curves and calibration curves to test the validity and robustness of the nomogram.

### Gene Ontology and Kyoto Encyclopedia of Genes and Genomes analysis

Eleven prognosis-related NRGs were annotated and functionally analyzed using the R “DOSE” package, including Gene Ontology (GO) and Kyoto Encyclopedia of Genes and Genomes (KEGG), with a corrected *p*-value (*q*-value) < 0.05 as the filter.

### Correlation between risk score and immune landscape

To reveal the correlation between risk scores and tumor-infiltrating immune cells, we assessed the immune infiltration of tumors using the CIBERSORT algorithm. We uploaded the full gene expression data of all samples to the CIBERSORTx portal and later ran the algorithm for 1,000 permutations based on the LM22 signature. LUAD samples with output *p*-values < 0.05 were selected for further analysis to explore the relationship between risk score and necroptosis-related prognostic gene expression and immune cell infiltration. The “estimate” software package was used to calculate the immune score and stromal score for each sample to quantify the relative enrichment of immune and stromal cells in each sample. Violin plots were applied to visualize the differences in enrichment between high and low-risk groups.

### Predicting patient response to immunotherapy

We further explored the potential role of risk scores in the prediction of immunotherapy using the immunophenoscore (IPS). Based on relevant data from The Cancer Imaging Archive (TCIA) database (https://www.cancerimagingarchive.net), we evaluated the differences in four IPS scores between high and low-risk groups. The scoring scheme integrates the four major classes of genes that determine tumor immunogenicity (effector cells, immunosuppressive cells, MHC molecules, and immunomodulators) and the gene expression of these cell types (e.g., activated CD4^+^ T cells, activated CD8^+^ T cells, effector memory CD4^+^ T cells, Tregs, MDSCs) to derive specific scores without bias using machine learning that is viewed as a new and reliable predictor of response to immunotherapy regimens (Givechian et al., 2018).

Tumor mutational burden (TMB) is broadly defined as the number of somatic mutations per megabase of interrogated genomic sequence. TMB reflects the total number of mutations carried by tumor cells. It is now generally accepted that TMB is positively correlated with the effect of immunotherapy and can be used as a potential molecular diagnostic marker for tumor immune checkpoint inhibitor therapy (Mayakonda et al., 2018). We obtained TMB information for the corresponding TCGA-LUAD cohort from the TCGA database. Spearman’s method was used for correlation analysis.

### Assessment of patients’ sensitivity to chemotherapy

With the “pRRophetic” software package, we reliably predicted the response to chemotherapy in each LUAD sample. The package works by using gene expression and drug sensitivity data from a very large panel of cancer cell lines in the Genomics of Drug Sensitivity in Cancer (GDSC) database (www.cancerrxgene.org/) as training data for developing statistical models (Yang et al., 2013). These models were then applied to gene expression data from other tumor biopsies to predict the clinical drug response of other samples to different anticancer drugs, with the half maximal inhibitory concentration (IC50) value of the target drug as the predicted outcome variable. The robustness of the model has been extensively validated (Geeleher et al., 2014a). After that, we reflected the difference in chemotherapy sensitivity between high and low-risk groups by box-line plots.

### Statistical methods

The study was statistically analyzed using R programming language (version 4.0.3). The Wilcoxon test was used to analyze continuous variables. Categorical variables were analyzed using Fisher’s exact test or Chi-square test. Survival differences were analyzed using Kaplan-Meier curves and log-ranch tests. *p*-values < 0.05 were considered statistically significant.

## Results

**FIGURE 1:**
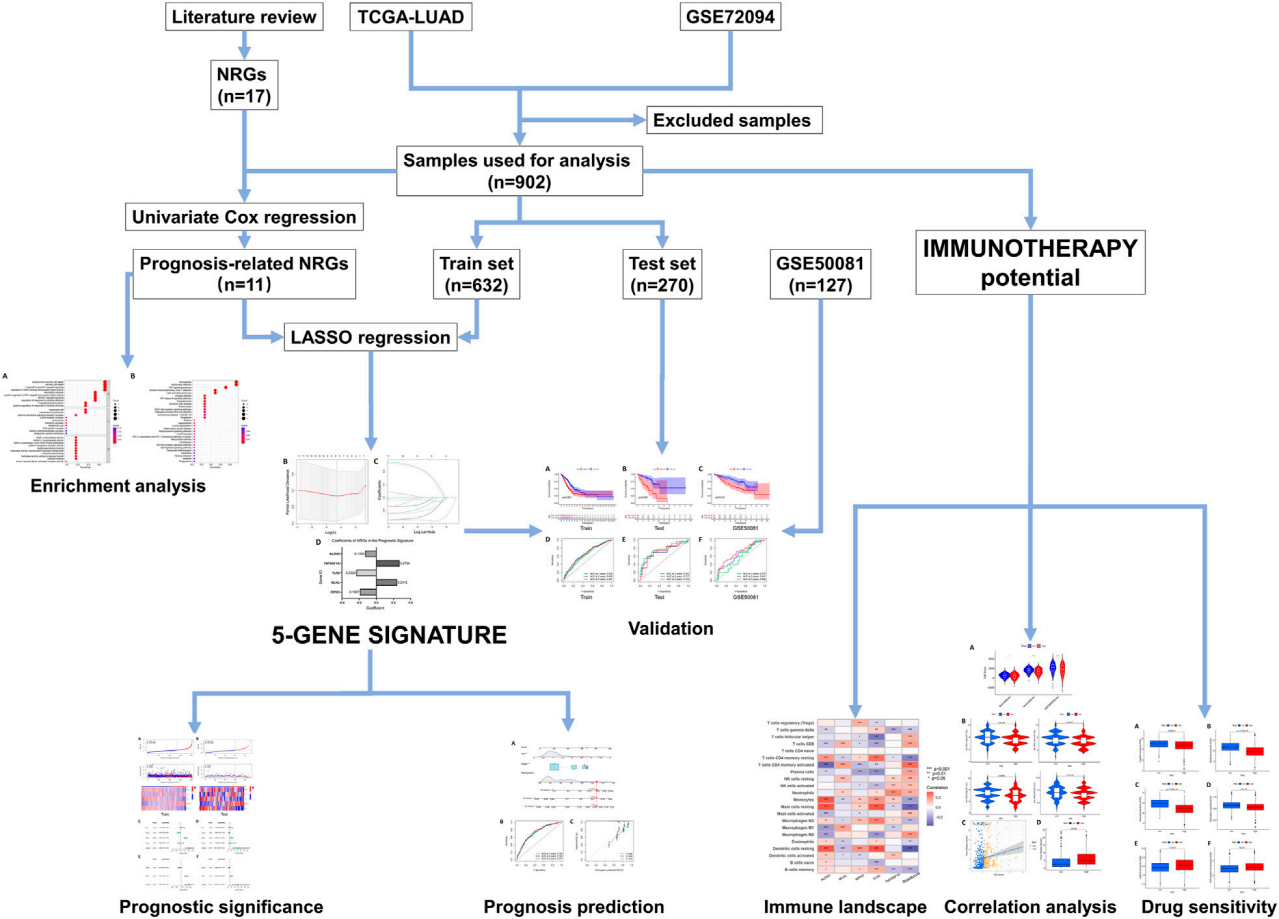
The framework and workflow of this study.

The design and workflow of this study are shown in Figure 1.

### Identification of necroptosis-related genes in lung adenocarcinoma

**FIGURE 2:**
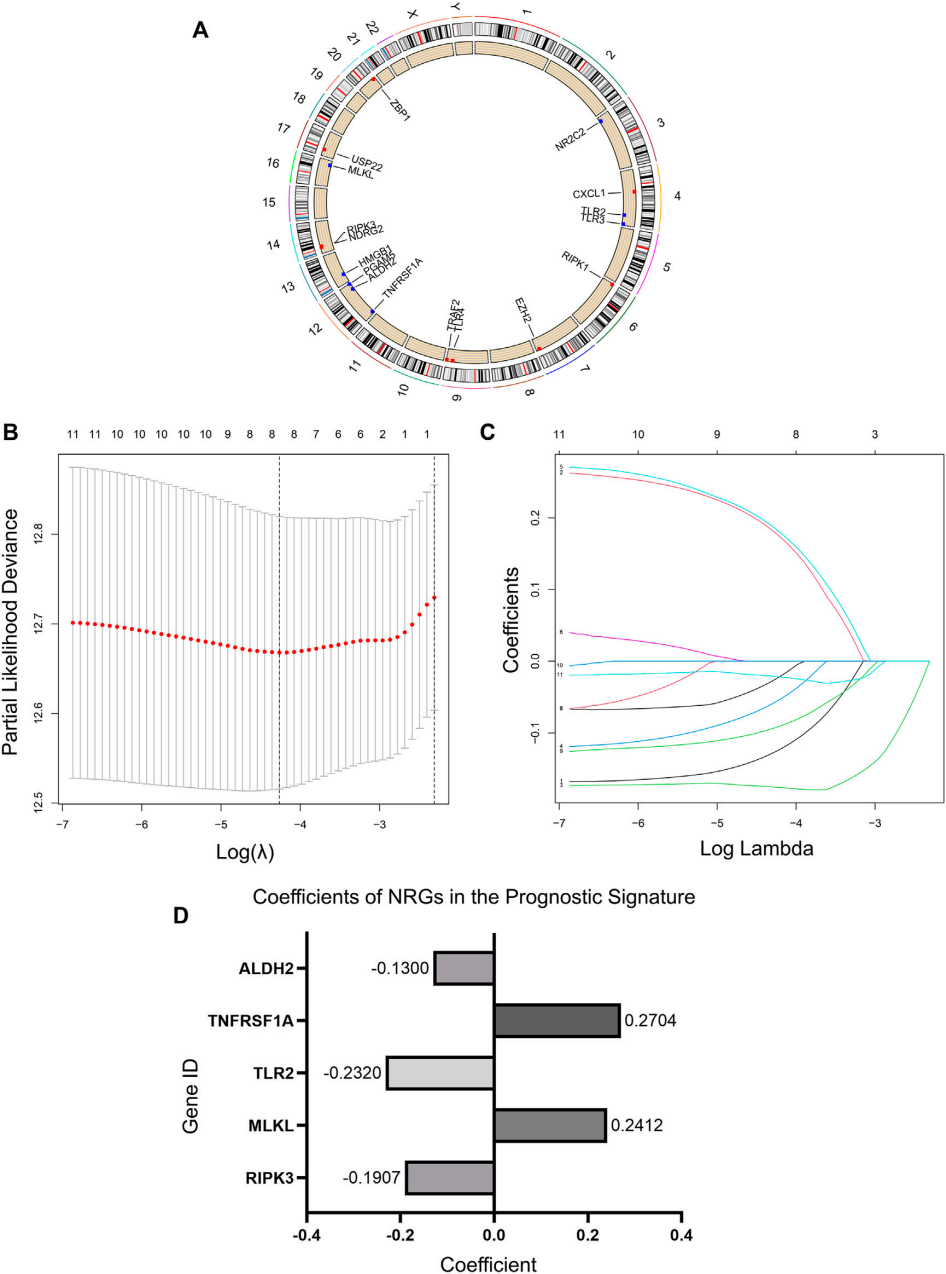
Necroptosis regulators in LUAD and an NRG signature. **(A)** Location of NRGs on chromosomes. **(B)** Ten‐fold cross‐validation for tuning parameter selection in the lasso regression. The vertical lines are plotted based on the optimal data according to the minimum criteria and 1-standard error criterion. The left vertical line represents the five genes finally identified. **(C)** LASSO coefficient profiles of 11 candidate genes and an optimal model derived from them. **(D)** 5 NRGs and their coefficients in the prognostic signature.

We obtained 17 NRGs (RIPK1, RIPK3, MLKL, TLR2, TLR3, TLR4, TNFRSF1A, PGAM5, ZBP1, NR2C2, HMGB1, CXCL1, USP22, TRAF2, ALDH2, EZH2, NDRG2) from previous literature reviews. The positions of these genes on the chromosomes are shown in Figure 2A. Immunohistochemical (IHC) staining results provided expression levels of 13 (RIPK1, TLR3, TLR4, TNFRSF1A, PGAM5, ZBP1, NR2C2, HMGB1, USP22, TRAF2, ALDH2, EZH2, NDRG2) of the 17 necroptotic proteins between LUAD and normal lung tissues (Supplementary Figure S1). For some reason, the remaining four genes could not be found in the HPA database with evidence of corresponding IHC staining.

By univariate Cox regression analysis of RNA transcriptome data, we identified 11 NRGs that were significantly associated with OS in LUAD patients. These genes and their HR, and *p*-values were listed in Table 1 and the clinical-pathological characteristics of 902 LUAD patients in TCGA + GSE72094 dataset were shown in Table 2.

**TABLE 1:** Univariate Cox analysis of prognostic NRGs.

ID	HR	HR.95L	HR.95H	*p*-value
RIPK3	0.775939	0.655868	0.917991	0.003101
MLKL	1.22469	1.01345	1.479959	0.035881
TLR2	0.786491	0.707495	0.874307	8.70E-06
TLR4	0.838561	0.736965	0.954162	0.007539
TNFRSF1A	1.534057	1.218781	1.930888	0.000267
PGAM5	1.513917	1.248294	1.836062	2.52E-05
NR2C2	0.794317	0.646461	0.97599	0.028436
TRAF2	1.313365	1.08852	1.584655	0.004437
ALDH2	0.74703	0.654351	0.852836	1.59E-05
EZH2	1.198274	1.0576	1.35766	0.004527
NDRG2	0.790279	0.696952	0.896103	0.000242

**TABLE 2:** The clinical characteristics of LUAD patients in the TCGA and GSE72094 datasets.

Clinical characteristics		Total	%
TCGA		504	100
Survival status	Alive	321	63.69
	Dead	183	36.31
Age	≥60 years old	358	71.03
	<60 years old	136	26.98
	Unknown	10	1.98
Gender	Male	234	46.43
	Female	270	53.57
Stage	I	270	53.57
	II	119	23.61
	III	81	16.07
	IV	26	5.16
	Unknown	8	1.59
T classification	T1	168	33.33
	T2	269	53.37
	T3	45	8.93
	T4	19	3.77
	Tx	3	0.60
N classification	N0	325	64.48
	N1	94	18.65
	N2	71	14.09
	N3	2	0.40
	Nx	12	2.38
M classification	M0	335	66.47
	M1	25	4.96
	Mx	144	28.57
Pharmaceutical therapy	YES	61	12.10
	NO	68	13.49
	Unknown	375	74.41
Radiation therapy	YES	61	12.10
	NO	71	14.09
	Unknown	372	73.81
Locoregional surgery	YES	9	1.78
	NO	96	19.05
	Unknown	399	79.17
Metastatic surgery	YES	20	3.97
	NO	78	15.48
	Unknown	406	80.55
GSE72094		398	100
Survival status	Alive	285	71.61
	Dead	113	28.39
Age	≥60 years old	340	85.43
	<60 years old	58	14.57
Gender	Male	176	44.22
	Female	222	55.78
Race	White	377	94.72
	Others	21	5.28
Stage	I	254	63.82
	II	67	16.83
	III	57	14.32
	IV	15	3.77
	Unknown	5	1.26

### Identification and validation of necroptosis-related gene prognostic signature

We randomized 902 patients included in the study into the Train and Test sets. Then LASSO regression analysis was performed in the Train set samples to construct a prognostic signature that included five NRGs (Figures 2B,C). These five genes and their correlation coefficients in the signature were shown in Figure 2D. Risk scores were calculated based on the expression profile data for all patients according to the formula provided by the model.

We divided each set into low-risk and high-risk groups bounded by the optimal cut-off values calculated by “CatPredi” package and then we plotted Kaplan-Meier survival curves and ROC curves separately for the Train set, Test set, and GSE50081 to verify the robustness of the model.

**FIGURE 3:**
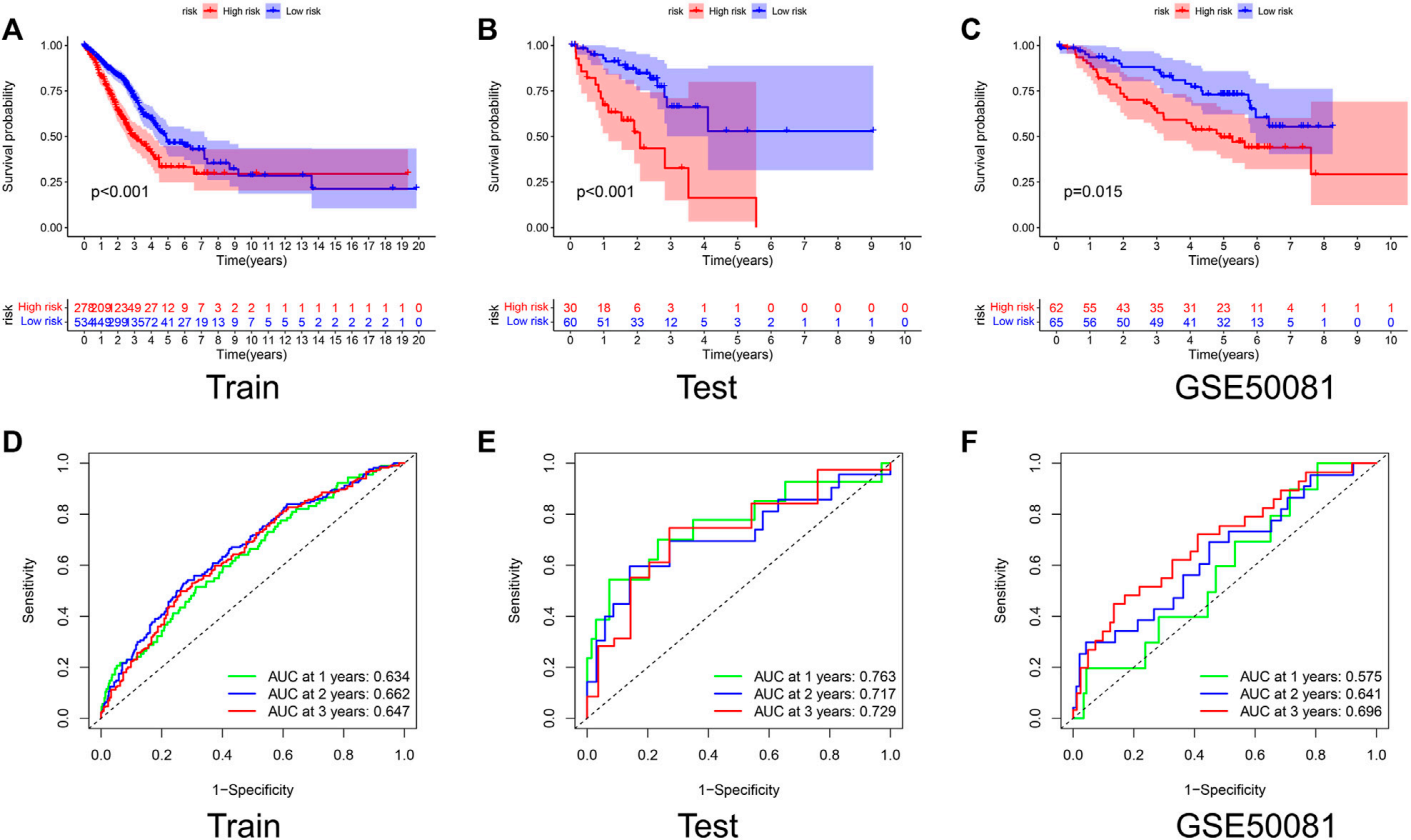
Validation of the prognostic signature. Kaplan–Meier curve presents differences in overall survival between the high-risk and low-risk groups in the Train set **(A)**, Test set **(B)**, and GSE50081 **(C)**. ROC curves of the NRG signature for predicting the 1/2/3-year survival in the Train set **(D)**, Test set **(E)**, and GSE50081 **(F)**. All results were statistically significant, demonstrating the good performance of the signature in assessing prognosis.

The K-M curves showed that the OS of the high-risk group is much lower than that of the low-risk group in both the Train and Test sets, with *p*-values of < 0.001 (Figures 3A,B). The AUC values for the Test set exceeded 0.7 in each of the first 3 years (Figures 3D,E). To further test the reliability of our model, we selected GSE50081 as external data for validation, and both results also showed that the label performed well in assessing prognosis (Figures 3C,F), with a survival curve of *p* = 0.015 and an AUC > 0.69 at year 3, demonstrating the good performance of the signature in assessing prognosis.

### Risk score has independent prognostic significance

**FIGURE 4:**
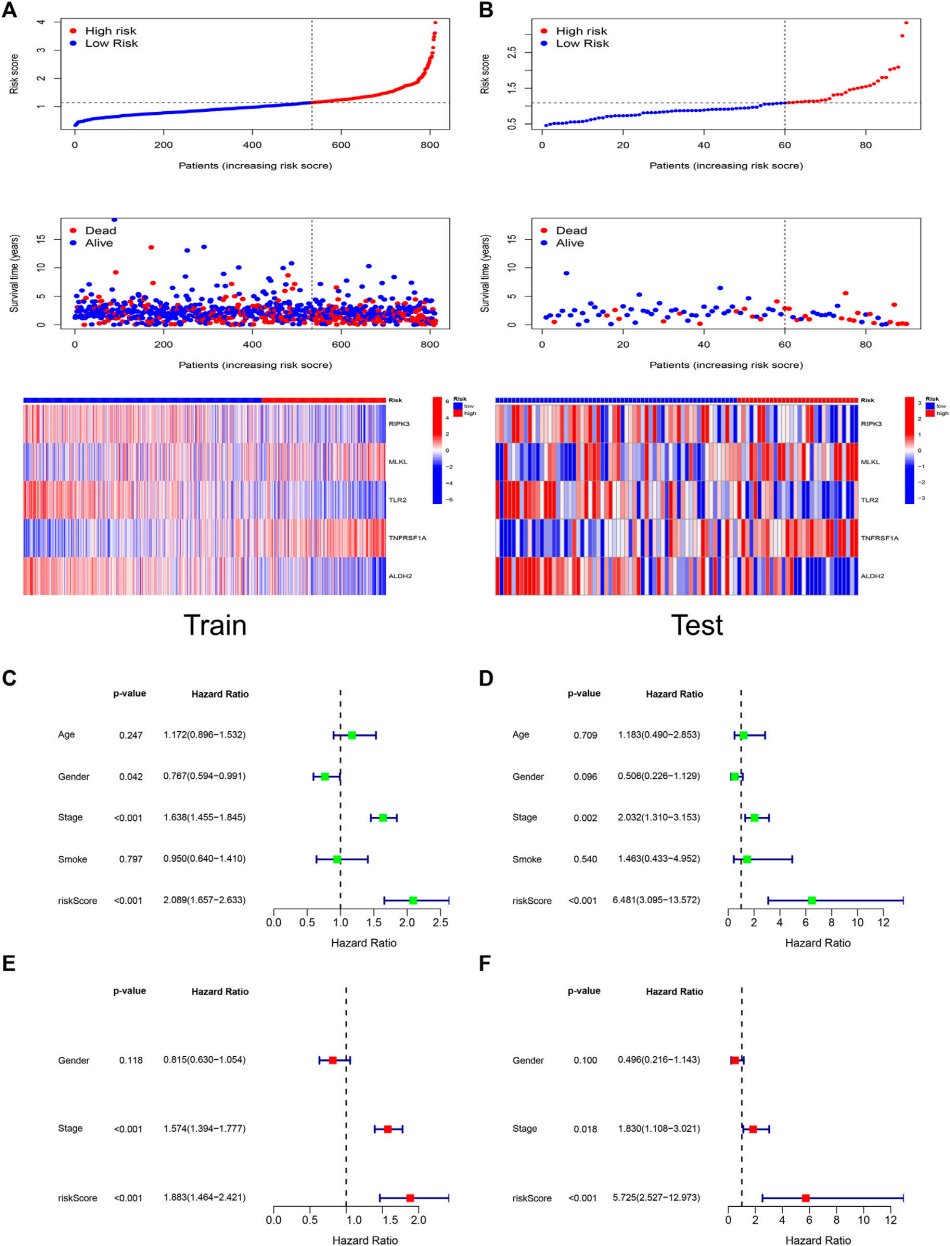
Risk score has independent prognostic significance. The trends of risk scores, the distribution of survival status, and the expression of the five genes included in the signature in Train set **(A)** and Test set **(B)**. The prognostic ability and clinical characteristics of the signature were analyzed by univariate Cox regression **(C)**, multivariate Cox regression **(E)** in Train set and validated by univariate Cox regression **(D)**, multivariate Cox regression **(F)** in Test set. The five-gene signature and stage were statistically significant in the Cox regression analysis.

Figures 4A,B demonstrated the relationship between risk score, patient survival, and gene expression of necroptosis regulators in the Train set and Test set, respectively. The heat map showed that RIPK3, TLR2, and ALDH2 were lowly expressed in the high-risk group, which corresponds to their correlation coefficients in the predictive signature (Figure 2D).

We performed Cox analyses to test whether the 5-gene signature was an independent predictor of OS in patients with LUAD. Univariate Cox regression analysis (Figures 4C,D) showed a significant association between risk score and OS. [Train set: HR 2.089, 95% confidence interval (CI) 1.657–2.633, *p* < 0.001; Test set: HR 6.481, 95% CI 3.095–13.572, *p* < 0.001]. After adjusting for other confounding variables, the five-gene signature remained an independent indicator of OS in multivariate Cox regression studies (Figures 4E,F) (Train set: HR 1.883, 95% CI 1.464–2.421, *p* < 0.001; Test set: HR 5.725, 95% CI 2.527–12.973, *p* < 0.001).

### Construction of a nomogram to quantitatively predict patient prognosis

**FIGURE 5:**
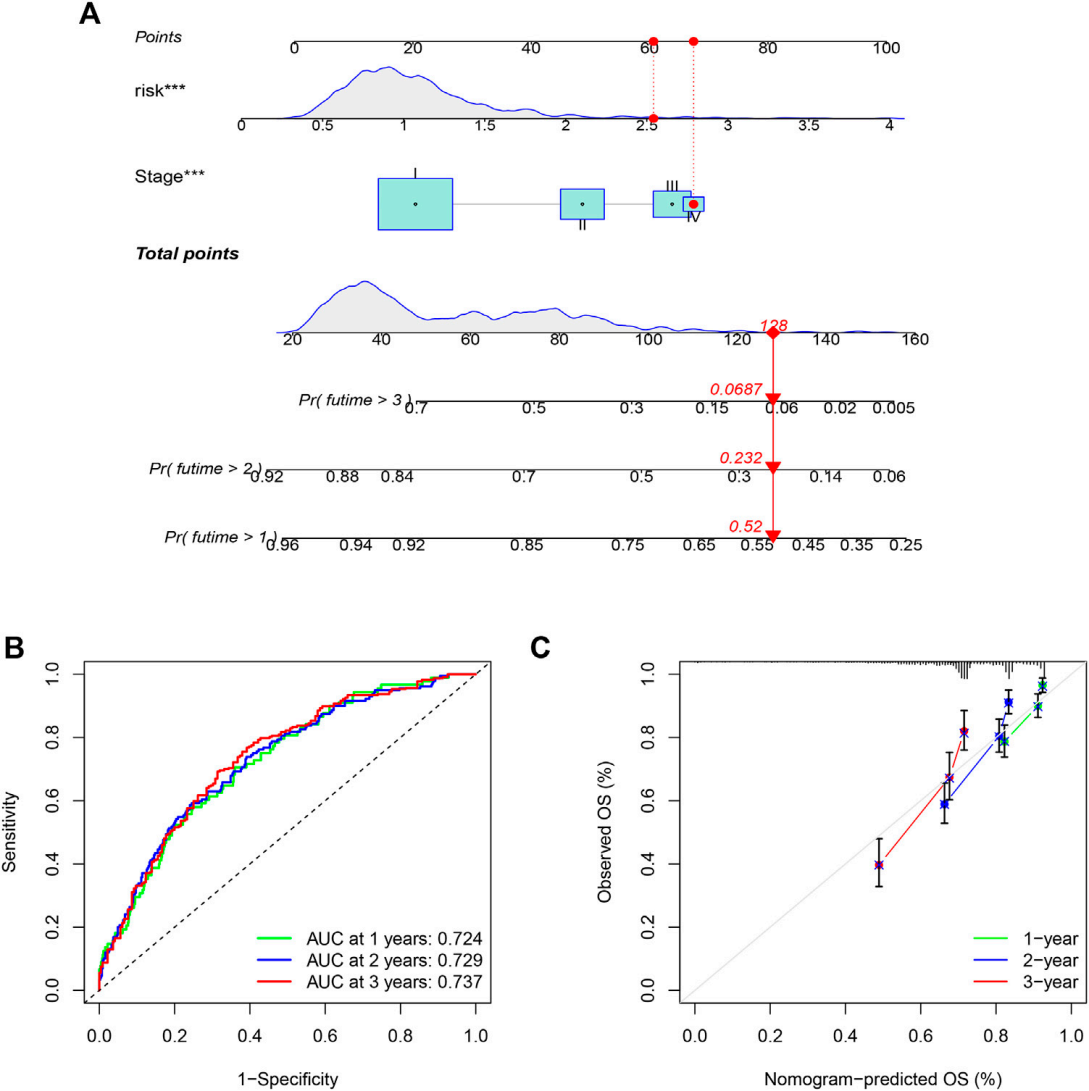
Nomogram was assembled by stage, and risk score for predicting the survival rate of LUAD patients **(A)**. ROC curves of the nomogram for predicting the 1/2/3-year survival **(B)**. 1/2/3-year nomogram calibration curves **(C)**. The results show that the nomogram model has very good predictive performance for prognosis (***p < 0.001).

To quantitatively predict the prognosis of LUAD patients, we developed a risk score-based nomogram (Figure 5A) that included tumor stages. The AUCs of curves at years 1, 2, and 3 were 0.724, 0.729, and 0.737, respectively (Figure 5B). Combined with the calibration curves of the nomogram shown in Figure 5C, the results show that the nomogram model has very good predictive performance for prognosis.

### Gene Ontology analysis and Kyoto Encyclopedia of Genes and Genomes analysis

**FIGURE 6:**
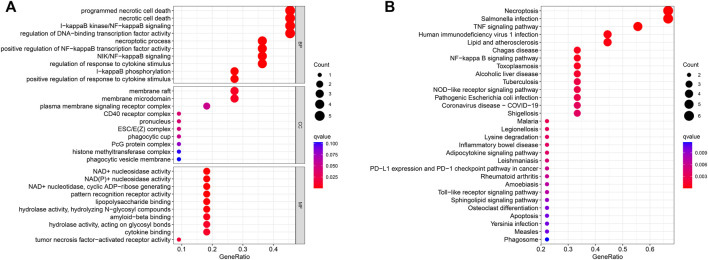
GO enrichment analysis **(A)** and KEGG enrichment analysis **(B)** of the 11 prognostic NRGs. The size of the circles in the graph indicates the number of genes enriched in the mechanism or pathway, and the color of the circles indicates the *q*-value.

To explore the preliminary function of the 11 prognosis-related NRGs, we did GO functional analysis and KEGG pathway enrichment analysis using the “ClusterProfiler” R package (adjusted *p* < 0.05, |logFC| > 1). GO analysis showed significant enrichment of genes in programmed necrotic cell death, necrotic cell death, I-kappaB kinase/NF-kappaB signaling, regulation of DNA-binding transcription factor activity, and other functions (Figure 6A). In KEGG pathway analysis, we learned that these genes are mainly concentrated in Necroptosis, Salmonella infection, and TNF signaling pathway (Figure 6B).

### Immune landscape and immunotherapy-related analysis

**FIGURE 7:**
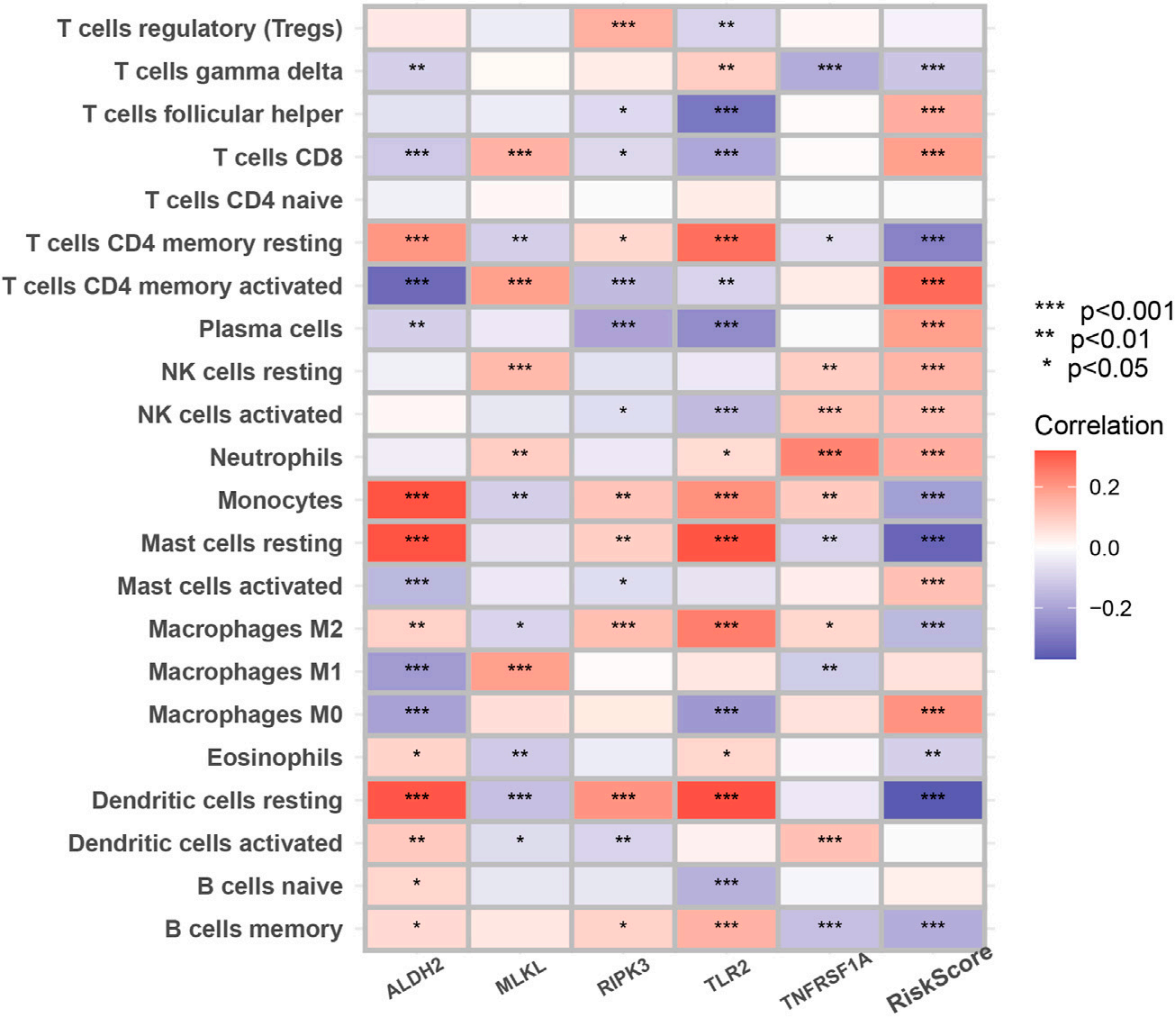
The correlation between infiltrating immune cells and NRGs in the signature. The change in the color of blocks implies the strength of the correlation (*p < 0.05; **p < 0.01; ***p < 0.001).

Figure 7 suggested that a variety of immune cells such as cytotoxic CD8^+^ T cells, Natural killer T cells, regulatory T cells, and macrophages are highly correlated with the risk score as well as key genes in the process of necroptosis and may play an important role in this prognostic signature.

**FIGURE 8:**
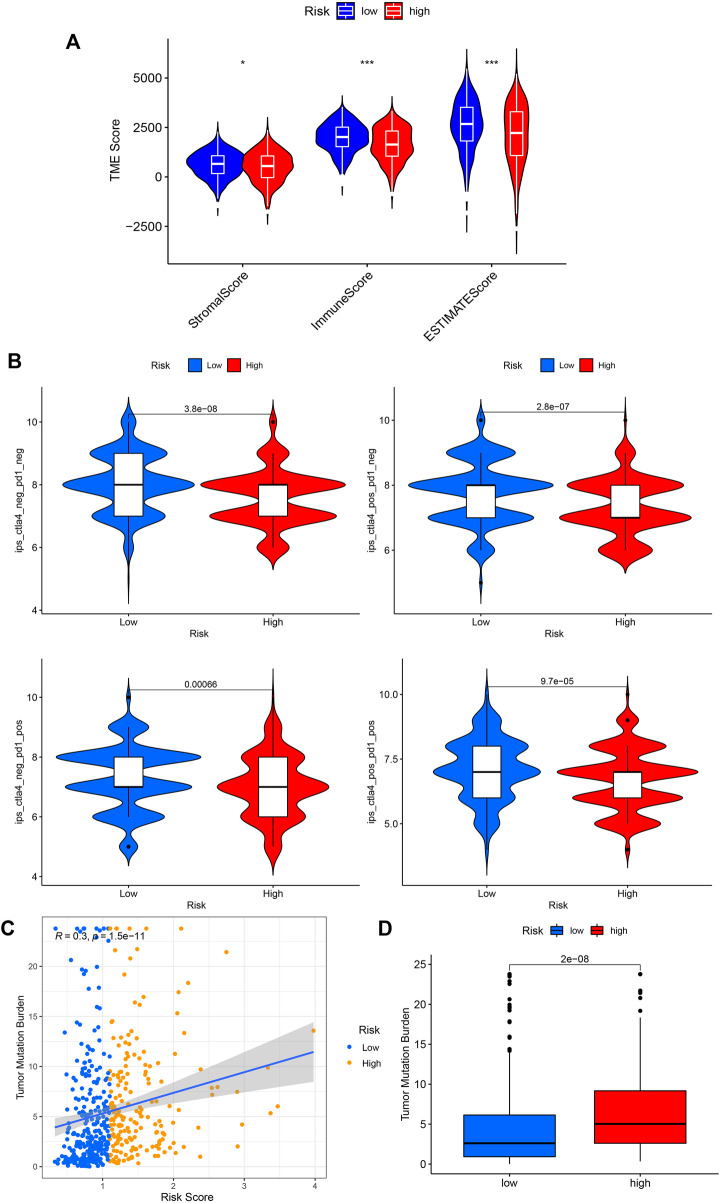
Application of risk score in predicting the immunotherapeutic effect. **(A)** Differential analysis of the tumor microenvironment for relative enrichment of immune cells and stromal cells. The low-risk group had a higher degree of immune cell infiltration and lower tumor purity. **(B)** The immunophenoscore (IPS) distribution was compared between high and low-risk groups. **(C,D)** Tumor Mutation Burden correlation analysis showed a positive correlation between risk score and TMB. (*p < 0.05; **p < 0.01; ***p < 0.001; pos means positive; neg means negative).

We then performed the ESTIMATE analysis (Figure 8A), and the immune score and ESTIMATE score were significantly lower in the high-risk group. Considering that patients in high and low-risk groups may respond differently to immunotherapy, we further investigated the response to ICI therapy represented by CTLA4/PD-1 inhibitors in both groups by ImmunoPhenoScore (IPS). Regardless of whether the CTLA4 and PD-1 status was positive or negative, patients in the low-risk group had higher IPS than those in the low-risk group, and the difference was statistically significant (Figure 8B).

Furthermore, we performed a TMB correlation analysis, Figures 8C,D, showing that the Tumor Mutation Burden differed significantly between the two groups of high and low risk according to the risk score. The risk score and TMB were positively correlated, *r* = 0.3.

### High-risk groups are more sensitive to chemotherapy

**FIGURE 9:**
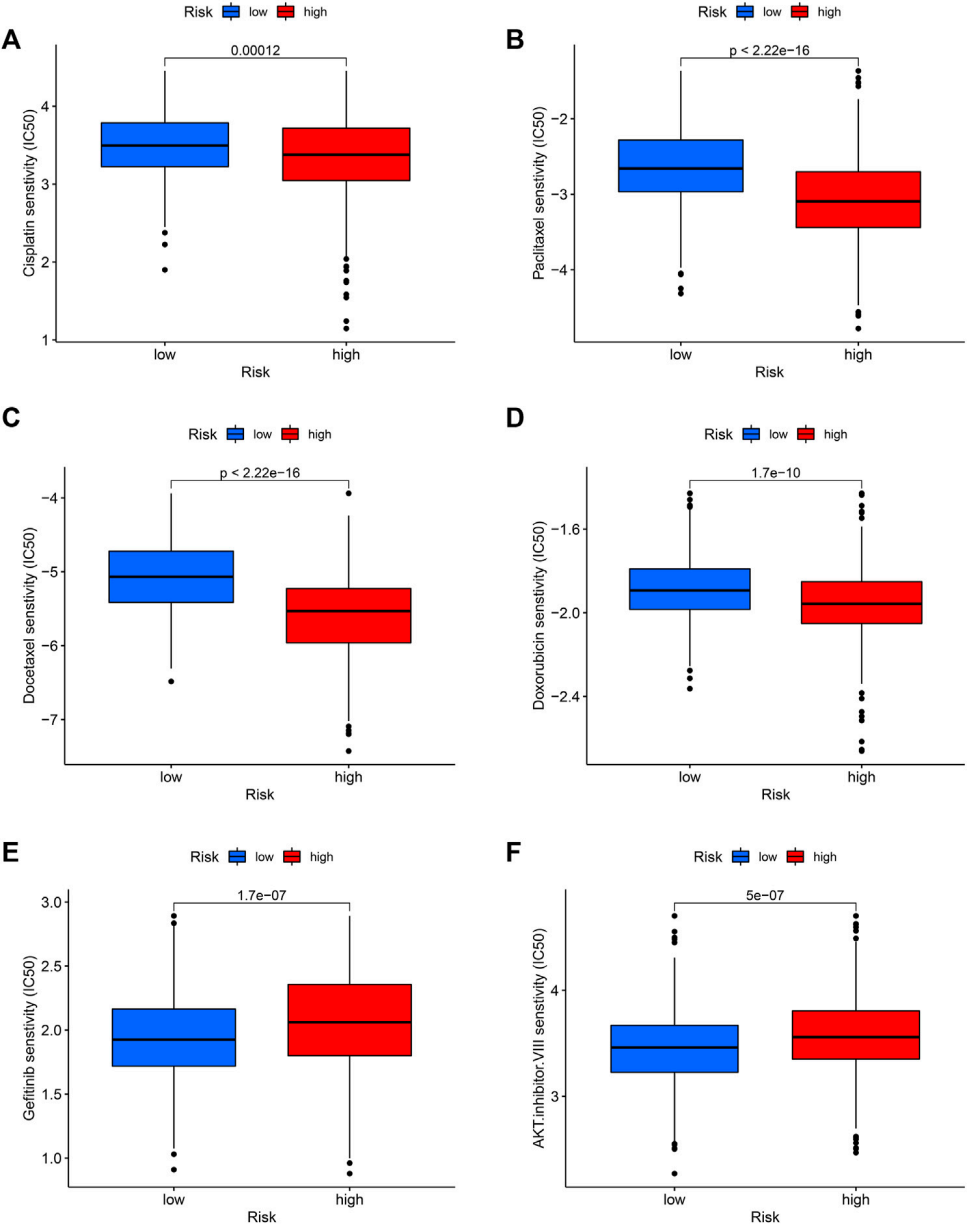
Potential drug sensitivity of common drugs in high and low-risk groups. The high-risk group had a higher sensitivity to chemotherapeutic agents such as cisplatin **(A)**, paclitaxel **(B)**, docetaxel **(C)**, and doxorubicin **(D)** compared to the low-risk group. However, the low-risk group was more sensitive to targeted therapeutic agents gefitinib **(E)** and AKT inhibitor VIII **(F)**.

Finally, we tested the sensitivity of patients in high and low-risk groups to familiar drugs based on a database of the GDSC. The R package “pRRophetic” (Geeleher et al., 2014a; Geeleher et al., 2014b) allows us to calculate IC50 for common chemotherapeutic agents in the cohort, including cisplatin, paclitaxel, and doxorubicin, rapamycin, etc. The lower the IC50 value, the higher the drug sensitivity. So, patients with LUAD in the high-risk group were significantly more sensitive to common chemotherapeutic agents such as cisplatin, paclitaxel, docetaxel, and doxorubicin (Figures 9A–D). Additionally, for the targeted drug of lung cancer, Gefitinib had a favorable response in the low-risk group (Figure 9E) and so did AKT inhibitor VIII (Figure 9F), suggesting that targeted therapy may provide benefit to these patients.

## Discussion

Necroptosis, as a form of programmed cell death, is a regulated form of necrosis. It has biological changes like that of cell necrosis, such as a dramatic increase in intracellular peroxides, highly phosphorylated mitochondrial membranes, and cell swelling, but the mechanisms of their initiation are not the same. Necrosis is defined as a non-programmed form of cell death characterized by cellular rupture. This allows the leakage of biomolecules such as damage-associated molecular patterns (DAMP), which are recognized by immune cells and trigger an inflammatory response. In contrast, necroptosis is controlled by a unique signaling pathway, which requires RIPK3-dependent MLKL phosphorylation. This phosphorylation event causes MLKL to produce a pore complex at the plasma membrane, which leads to DAMP secretion, cell swelling, and membrane rupture.

In this study, we constructed a prognostic signature associated with NRGs and demonstrated its good and accurate prognostic prediction ability using a combination of internal validation (Test set) and external validation (GSE50081). Cox regression analysis showed that our risk score could be used as a strong predictor of prognosis for LUAD patients. Interestingly, the fact that smoking was not an independent prognostic factor for patients was also confirmed in our study. According to Jemal et al. (2018), an increasing proportion of patients diagnosed with lung cancer are non-smokers, especially among those diagnosed with LUAD, despite that smoking has long been recognized as one of the important risk factors for lung cancer (Gould et al., 2013).

A total of five NRGs were included in the prognostic prediction model, which were RIPK3, MLKL, TLR2, TNFRSF1A, and ALDH2. Several studies have been conducted to explore the function of these genes, especially the relationship between these genes and cancer. RIPK3 is thought to be a key molecular switch for the initiation of necroptosis in cells. In the classical necroptotic pathway, deubiquitinated RIPK1 interacts with RIPl3 *via* exposure of the RIP homotypic interaction motif (RHIM) structural domain and conformational changes to phosphorylate and form amyloid signaling complex necrosomes together with FADD/caspase8 (Anderton et al., 2019). Then, MLKL is recruited and phosphorylated to mediate the execution of necroptosis. When RIPK1 is deficient, DNA-dependent activator of interferon regulatory factors (DAI), lipopolysaccharide, and chemical inducers can directly activate RIPK3 through a non-caspase-dependent mechanism, and activated RIPK3 phosphorylates MLKL to mediate necrosis signaling (Brault and Oberst, 2017). Multiple cancers suppress necroptosis through epigenetic silencing of RIPK3, which is consistent with our obtained finding that the mRNA expression of RIPK3 in the model is negatively correlated with the patient’s risk score.

Although activation of MLKL is the executor of the necroptotic process, the expression of MLKL varies much across cancers, which is related to its complex cytological function. Recent studies have revealed that MLKL has an important role in a variety of non-necroptotic processes such as axonal repair, receptor internalization, extracellular vesicle formation, ligand-receptor degradation, and even in the inhibition of necroptosis (Martens et al., 2021). Unfortunately, the exact role of MLKL in cancer progression and metastasis is still unclear.

TLR2 induces TNF expression mainly through the Myeloid differentiation factor 88 (MyD88)-dependent pathway, which can indirectly trigger apoptosis or triggers the classical pathway of necroptosis through RIP1-RIP3 activation (Kaiser et al., 2013). Interestingly, TNF is generally recognized to inhibit or kill tumor cells through multiple links, TNF receptors (TNFR), especially TNFR1, have been found to be upregulated in a variety of tumors, such as ovarian cancer (Le Page et al., 2006), renal clear cell carcinoma (Diegmann et al., 2006) and acute myeloid leukemia (Brouwer et al., 2001). This may be related to the fact that TNFR1, in addition to being involved in mediating apoptosis and necroptosis, can also mediate cell activation signals and proliferation signals that drive the expression of pro-survival genes (Dondelinger et al., 2016). TNFRSF1A, the gene encoding the TNFR1 protein, was similarly found to be highly expressed in the high-risk group of LUAD patients in our present study. ALDH2 belongs to the acetaldehyde dehydrogenase family, and its reduction not only induces proliferation and stem cell properties of LUAD cells but also may induce DNA damage, which will promote tumor recurrence, drug resistance, and metastasis, leading to poor prognosis of LUAD (Li et al., 2019).

Many studies have shown that cancer cells undergoing necroptosis mediate immune responses by promoting interactions between dying cancer cells and immune cells through the release of damage-associated molecular patterns (DAMPs), chemokines, cytokines, and/or cancer antigens (Krieser and White, 2002; Park et al., 2009; McCracken et al., 2015; Sprooten et al., 2020). In terms of tumor suppression, DAMPs can eliminate cancer cells by stimulating the initial sensors of infection or damage (e.g., pattern recognition receptors on myeloid cells) and activating adaptive immune cells such as antigen-specific cytotoxic CD8^+^ T cells (Yatim et al., 2015). A significant positive correlation of MLKL with cytotoxic CD8^+^ T cells was also demonstrated in our study. In addition to this, RIPK3 can induce the production of cytokines to activate natural killer T cells, which also help to kill cancer cells (Sprooten et al., 2020). However, regarding tumor promotion, necroptotic cancer cells can also attract myeloid or lymphocytes, triggering tumor-associated immunosuppression (Cohen et al., 2010; Vandenberk et al., 2016; Wauters et al., 2021). For example, in Figure 7, RIPK3 exhibited a significant positive correlation with regulatory T cell and Macrophages M2. Besides, cytokines released from necrotic apoptotic cancer cells can promote angiogenesis, reactive oxygen species release, and genomic instability, thus promoting tumor progression (Singh et al., 2017).

Furthermore, in addition to these biological insights, our study plays an important role in guiding the use of immunotherapy in patients with LUAD. The background features of the immunobiology of necroptosis, combined with a more complex tumor immune landscape, can produce highly unpredictable outcomes for immunotherapy of tumors. ICIs do greatly improve the prognosis of cancer patients, but a minority still does not respond adequately to these immunotherapies, as treatment efficacy is largely influenced by immune cell abundance, tumor mutation burden, and other biomolecules (He et al., 2015; Sharma et al., 2017; Herbst et al., 2018). Combined results, our study has important implications for the use of single ICIs as well as combined ICIs in patients with LUAD. Therefore, it will be interesting to systematically decipher whether ICI-based immunotherapy can synergize with necroptosis and produce unknown benefits. More importantly, we predicted the sensitivity of chemotherapeutic agents, which helps physicians choose the right combination of chemotherapy and immunotherapy to improve the survival rate of LUAD patients. This is because the efficacy of ICIs can be greatly improved when co-administered with cytotoxic therapy (Judd and Borghaei, 2020).

Of course, our study has many drawbacks. Firstly, necroptosis is a new and rapidly developing field, and more and more NRGs will be discovered and fully studied over time. Our findings will be fleshed out then. Secondly, all data samples in this study were obtained from public open-source databases. Due to the relatively small number of LUAD patients in public databases and the duplication of transcriptome data in different databases, the sample size covered in the randomized grouping of this study was relatively insufficient, resulting in the low significance of some results. On the other hand, some important clinical details were not available in the open-source dataset, including chemotherapy regimens, drug information, and tumor TNM grading. And the lack of these data limits more in-depth analysis of the dataset. In addition, some of the prognostic NRG-associated immunohistochemical slides in the HPA database were not available, which also left us with a regret for our study. Finally, the role of some NRGs in non-small cell lung cancer is unclear and still needs to be revealed by further *in vivo* or *in vitro* experiments.

In conclusion, we constructed a robust NRG-related prognostic signature that could be used to predict the prognosis of LUAD patients. We also analyzed the sensitivity of different immunotherapy and chemotherapy regimens, which could provide a reference to improve patient prognosis and achieve personalized medicine. Meanwhile, we believe that this study provides insight into the potential role of necroptosis in lung adenocarcinoma.

## Data Availability

The original contributions presented in the study are included in the article/Supplementary Material, further inquiries can be directed to the corresponding authors.

## Supplementary material

SUPPLEMENTARY FIGURE S1 Immunohistochemical (IHC) staining results of expression levels of the 13 necroptotic proteins (RIPK1, TLR3, TLR4, TNFRSF1A, PGAM5, ZBP1, NR2C2, HMGB1, USP22, TRAF2, ALDH2, EZH2, NDRG2) between LUAD and normal lung tissues.

## References

[B1] Anderton H. Bandala-Sanchez E. Simpson D. S. Rickard J. A. Ng A. P. Di Rago L. (2019). RIPK1 prevents TRADD-driven, but TNFR1 independent, apoptosis during development. Cell Death Differ. 26 (5), 877–889. 10.1038/s41418-018-0166-8 30185824 PMC6461919

[B2] Bonavida B. Chouaib S. (2017). Resistance to anticancer immunity in cancer patients: Potential strategies to reverse resistance. Ann. Oncol. 28 (3), 457–467. 10.1093/annonc/mdw615 27864216 PMC5834050

[B3] Brault M. Oberst A. (2017). Controlled detonation: Evolution of necroptosis in pathogen defense. Immunol. Cell Biol. 95 (2), 131–136. 10.1038/icb.2016.117 27909314 PMC6855669

[B4] Brouwer R. E. Hoefnagel J. Borger van Der Burg B. Jedema I. Zwinderman K. H. Starrenburg I. C. (2001). Expression of co-stimulatory and adhesion molecules and chemokine or apoptosis receptors on acute myeloid leukaemia: High CD40 and CD11a expression correlates with poor prognosis. Br. J. Haematol. 115 (2), 298–308. 10.1046/j.1365-2141.2001.03085.x 11703324

[B5] Chen Z. Fillmore C. M. Hammerman P. S. Kim C. F. Wong K. K. (2014). Non-small-cell lung cancers: A heterogeneous set of diseases. Nat. Rev. Cancer 14 (8), 535–546. 10.1038/nrc3775 25056707 PMC5712844

[B6] Cheng M. Lin N. Dong D. Ma J. Su J. Sun L. (2021). PGAM5: A crucial role in mitochondrial dynamics and programmed cell death. Eur. J. Cell Biol. 100 (1), 151144. 10.1016/j.ejcb.2020.151144 33370650

[B7] Choi M. E. Price D. R. Ryter S. W. Choi A. M. K. (2019). Necroptosis: A crucial pathogenic mediator of human disease. JCI Insight 4 (15), 128834. 10.1172/jci.insight.128834 31391333 PMC6693822

[B8] Cohen I. Rider P. Carmi Y. Braiman A. Dotan S. White M. R. (2010). Differential release of chromatin-bound IL-1alpha discriminates between necrotic and apoptotic cell death by the ability to induce sterile inflammation. Proc. Natl. Acad. Sci. U. S. A. 107 (6), 2574–2579. 10.1073/pnas.0915018107 20133797 PMC2823886

[B9] Degterev A. Huang Z. Boyce M. Li Y. Jagtap P. Mizushima N. (2005). Chemical inhibitor of nonapoptotic cell death with therapeutic potential for ischemic brain injury. Nat. Chem. Biol. 1 (2), 112–119. 10.1038/nchembio711 16408008

[B10] Devarakonda S. Morgensztern D. Govindan R. (2015). Genomic alterations in lung adenocarcinoma. Lancet. Oncol. 16 (7), e342–e351. 10.1016/S1470-2045(15)00077-7 26149886

[B11] Diegmann J. Tomiuk S. Sanjmyatav J. Junker K. Hindermann W. Von Eggeling F. (2006). Comparative transcriptional and functional profiling of clear cell and papillary renal cell carcinoma. Int. J. Mol. Med. 18 (3), 395–403. 10.3892/ijmm.18.3.395 16865223

[B12] Dondelinger Y. Darding M. Bertrand M. J. Walczak H. (2016). Poly-ubiquitination in TNFR1-mediated necroptosis. Cell. Mol. Life Sci. 73 (11-12), 2165–2176. 10.1007/s00018-016-2191-4 27066894 PMC4887548

[B13] Geeleher P. Cox N. Huang R. S. (2014). pRRophetic: an R package for prediction of clinical chemotherapeutic response from tumor gene expression levels. PLoS One 9 (9), e107468. 10.1371/journal.pone.0107468 25229481 PMC4167990

[B14] Geeleher P. Cox N. J. Huang R. S. (2014). Clinical drug response can be predicted using baseline gene expression levels and *in vitro* drug sensitivity in cell lines. Genome Biol. 15 (3), R47. 10.1186/gb-2014-15-3-r47 24580837 PMC4054092

[B15] Givechian K. B. Wnuk K. Garner C. Benz S. Garban H. Rabizadeh S. (2018). Identification of an immune gene expression signature associated with favorable clinical features in Treg-enriched patient tumor samples. NPJ Genom. Med. 3, 14. 10.1038/s41525-018-0054-7 29928512 PMC5998068

[B16] Gong Y. Fan Z. Luo G. Yang C. Huang Q. Fan K. (2019). The role of necroptosis in cancer biology and therapy. Mol. Cancer 18 (1), 100. 10.1186/s12943-019-1029-8 31122251 PMC6532150

[B17] Gould M. K. Donington J. Lynch W. R. Mazzone P. J. Midthun D. E. Naidich D. P. (2013). Evaluation of individuals with pulmonary nodules: When is it lung cancer? Diagnosis and management of lung cancer, 3rd ed: American college of chest physicians evidence-based clinical practice guidelines. Chest 143 (5), e93S–e120S. 10.1378/chest.12-2351 23649456 PMC3749714

[B18] Hänggi K. Vasilikos L. Valls A. F. Yerbes R. Knop J. Spilgies L. M. (2017). RIPK1/RIPK3 promotes vascular permeability to allow tumor cell extravasation independent of its necroptotic function. Cell Death Dis. 8 (2), e2588. 10.1038/cddis.2017.20 28151480 PMC5386469

[B19] He J. Hu Y. Hu M. Li B. (2015). Development of PD-1/PD-L1 pathway in tumor immune microenvironment and treatment for non-small cell lung cancer. Sci. Rep. 5, 13110. 10.1038/srep13110 26279307 PMC4538573

[B20] Herbst R. S. Morgensztern D. Boshoff C. (2018). The biology and management of non-small cell lung cancer. Nature 553 (7689), 446–454. 10.1038/nature25183 29364287

[B21] Jemal A. Miller K. D. Ma J. Siegel R. L. Fedewa S. A. Islami F. (2018). Higher lung cancer incidence in young women than young men in the United States. N. Engl. J. Med. 378 (21), 1999–2009. 10.1056/NEJMoa1715907 29791813 PMC7717174

[B22] Judd J. Borghaei H. (2020). Combining immunotherapy and chemotherapy for non-small cell lung cancer. Thorac. Surg. Clin. 30 (2), 199–206. 10.1016/j.thorsurg.2020.01.006 32327178

[B23] Kaiser W. J. Sridharan H. Huang C. Mandal P. Upton J. W. Gough P. J. (2013). Toll-like receptor 3-mediated necrosis via TRIF, RIP3, and MLKL. J. Biol. Chem. 288 (43), 31268–31279. 10.1074/jbc.M113.462341 24019532 PMC3829437

[B24] Khoury M. K. Gupta K. Franco S. R. Liu B. (2020). Necroptosis in the pathophysiology of disease. Am. J. Pathol. 190 (2), 272–285. 10.1016/j.ajpath.2019.10.012 31783008 PMC6983729

[B25] Krieser R. J. White K. (2002). Engulfment mechanism of apoptotic cells. Curr. Opin. Cell Biol. 14 (6), 734–738. 10.1016/s0955-0674(02)00390-3 12473347

[B26] Le Page C. Ouellet V. Madore J. Ren F. Hudson T. J. Tonin P. N. (2006). Gene expression profiling of primary cultures of ovarian epithelial cells identifies novel molecular classifiers of ovarian cancer. Br. J. Cancer 94 (3), 436–445. 10.1038/sj.bjc.6602933 16421595 PMC2361148

[B27] Li K. Guo W. Li Z. Wang Y. Sun B. Xu D. (2019). ALDH2 repression promotes lung tumor progression via accumulated acetaldehyde and DNA damage. Neoplasia 21 (6), 602–614. 10.1016/j.neo.2019.03.008 31071657 PMC6506700

[B28] Liu X. Zhou M. Mei L. Ruan J. Hu Q. Peng J. (2016). Key roles of necroptotic factors in promoting tumor growth. Oncotarget 7 (16), 22219–22233. 10.18632/oncotarget.7924 26959742 PMC5008357

[B29] Lou X. Zhu H. Ning L. Li C. Li S. Du H. (2019). EZH2 regulates intestinal inflammation and necroptosis through the JNK signaling pathway in intestinal epithelial cells. Dig. Dis. Sci. 64 (12), 3518–3527. 10.1007/s10620-019-05705-4 31273598

[B30] Malireddi R. K. S. Kesavardhana S. Kanneganti T. D. (2019). ZBP1 and TAK1: Master regulators of NLRP3 inflammasome/pyroptosis, apoptosis, and necroptosis (PAN-optosis). Front. Cell. Infect. Microbiol. 9, 406. 10.3389/fcimb.2019.00406 31850239 PMC6902032

[B31] Martens S. Bridelance J. Roelandt R. Vandenabeele P. Takahashi N. (2021). MLKL in cancer: More than a necroptosis regulator. Cell Death Differ. 28 (6), 1757–1772. 10.1038/s41418-021-00785-0 33953348 PMC8184805

[B32] Matter M. S. Chijioke O. Savic S. Bubendorf L. (2020). Narrative review of molecular pathways of kinase fusions and diagnostic approaches for their detection in non-small cell lung carcinomas. Transl. Lung Cancer Res. 9 (6), 2645–2655. 10.21037/tlcr-20-676 33489824 PMC7815372

[B33] Mayakonda A. Lin D. C. Assenov Y. Plass C. Koeffler H. P. (2018). Maftools: Efficient and comprehensive analysis of somatic variants in cancer. Genome Res. 28 (11), 1747–1756. 10.1101/gr.239244.118 30341162 PMC6211645

[B34] McCracken M. N. Cha A. C. Weissman I. L. (2015). Molecular pathways: Activating T cells after cancer cell phagocytosis from blockade of CD47 "don't eat me" signals. Clin. Cancer Res. 21 (16), 3597–3601. 10.1158/1078-0432.CCR-14-2520 26116271 PMC4621226

[B35] Park S. Hatanpaa K. J. Xie Y. Mickey B. E. Madden C. J. Raisanen J. M. (2009). The receptor interacting protein 1 inhibits p53 induction through NF-kappaB activation and confers a worse prognosis in glioblastoma. Cancer Res. 69 (7), 2809–2816. 10.1158/0008-5472.CAN-08-4079 19339267 PMC2859885

[B36] Petersen S. L. Chen T. T. Lawrence D. A. Marsters S. A. Gonzalvez F. Ashkenazi A. (2015). TRAF2 is a biologically important necroptosis suppressor. Cell Death Differ. 22 (11), 1846–1857. 10.1038/cdd.2015.35 25882049 PMC4648330

[B37] Philipp S. Sosna J. Adam D. (2016). Cancer and necroptosis: Friend or foe? Cell. Mol. Life Sci. 73 (11-12), 2183–2193. 10.1007/s00018-016-2193-2 27048810 PMC11108265

[B38] Raposo T. P. Beirão B. C. Pang L. Y. Queiroga F. L. Argyle D. J. (2015). Inflammation and cancer: Till death tears them apart. Vet. J. 205 (2), 161–174. 10.1016/j.tvjl.2015.04.015 25981934

[B39] Roedig J. Kowald L. Juretschke T. Karlowitz R. Ahangarian Abhari B. Roedig H. (2021). USP22 controls necroptosis by regulating receptor-interacting protein kinase 3 ubiquitination. EMBO Rep. 22 (2), e50163. 10.15252/embr.202050163 33369872 PMC7857539

[B40] Rosenberg S. A. (2014). Decade in review-cancer immunotherapy: Entering the mainstream of cancer treatment. Nat. Rev. Clin. Oncol. 11 (11), 630–632. 10.1038/nrclinonc.2014.174 25311350 PMC6310157

[B41] Santarpia M. Aguilar A. Chaib I. Cardona A. F. Fancelli S. Laguia F. (2020). Non-small-cell lung cancer signaling pathways, metabolism, and PD-1/PD-L1 antibodies. Cancers (Basel) 12 (6), E1475. 10.3390/cancers12061475 PMC7352732 32516941

[B42] Sharma P. Hu-Lieskovan S. Wargo J. A. Ribas A. (2017). Primary, adaptive, and acquired resistance to cancer immunotherapy. Cell 168 (4), 707–723. 10.1016/j.cell.2017.01.017 28187290 PMC5391692

[B43] Singh R. Mishra M. K. Aggarwal H. (2017). Inflammation, immunity, and cancer. Mediat. Inflamm. 2017, 6027305. 10.1155/2017/6027305 PMC5695028 29234189

[B44] Sprooten J. De Wijngaert P. Vanmeerbeerk I. Martin S. Vangheluwe P. Schlenner S. (2020). Necroptosis in immuno-oncology and cancer immunotherapy. Cells 9 (8), E1823. 10.3390/cells9081823 PMC7464343 32752206

[B45] Sung H. Ferlay J. Siegel R. L. Laversanne M. Soerjomataram I. Jemal A. (2021). Global cancer statistics 2020: GLOBOCAN estimates of incidence and mortality worldwide for 36 cancers in 185 countries. Ca. Cancer J. Clin. 71 (3), 209–249. 10.3322/caac.21660 33538338

[B46] Tang R. Xu J. Zhang B. Liu J. Liang C. Hua J. (2020). Ferroptosis, necroptosis, and pyroptosis in anticancer immunity. J. Hematol. Oncol. 13 (1), 110. 10.1186/s13045-020-00946-7 32778143 PMC7418434

[B47] Vandenberk L. Garg A. D. Verschuere T. Koks C. Belmans J. Beullens M. (2016). Irradiation of necrotic cancer cells, employed for pulsing dendritic cells (DCs), potentiates DC vaccine-induced antitumor immunity against high-grade glioma. Oncoimmunology 5 (2), e1083669. 10.1080/2162402X.2015.1083669 27057467 PMC4801426

[B48] Wauters E. Van Mol P. Garg A. D. Jansen S. Van Herck Y. Vanderbeke L. (2021). Discriminating mild from critical COVID-19 by innate and adaptive immune single-cell profiling of bronchoalveolar lavages. Cell Res. 31 (3), 272–290. 10.1038/s41422-020-00455-9 33473155 PMC8027624

[B49] Wen S. Li X. Ling Y. Chen S. Deng Q. Yang L. (2020). HMGB1-associated necroptosis and Kupffer cells M1 polarization underlies remote liver injury induced by intestinal ischemia/reperfusion in rats. Faseb J. 34 (3), 4384–4402. 10.1096/fj.201900817R 31961020

[B50] Xia X. Lei L. Wang S. Hu J. Zhang G. (2020). Necroptosis and its role in infectious diseases. Apoptosis 25 (3-4), 169–178. 10.1007/s10495-019-01589-x 31912263

[B51] Yang W. Soares J. Greninger P. Edelman E. J. Lightfoot H. Forbes S. (2013). Genomics of drug sensitivity in cancer (GDSC): A resource for therapeutic biomarker discovery in cancer cells. Nucleic Acids Res. 41, D955–D961. 10.1093/nar/gks1111 23180760 PMC3531057

[B52] Yatim N. Jusforgues-Saklani H. Orozco S. Schulz O. Barreira da Silva R. Reis e Sousa C. (2015). RIPK1 and NF-κB signaling in dying cells determines cross-priming of CD8⁺ T cells. Science 350 (6258), 328–334. 10.1126/science.aad0395 26405229 PMC4651449

[B53] Zhu J. Yang L. K. Wang Q. H. Lin W. Feng Y. Xu Y. P. (2020). NDRG2 attenuates ischemia-induced astrocyte necroptosis via the repression of RIPK1. Mol. Med. Rep. 22 (4), 3103–3110. 10.3892/mmr.2020.11421 32945444 PMC7453600

