# Supplementary figures and images for: Prognostic necroptosis-related gene signature aids immunotherapy in lung adenocarcinoma
Source: Front Genet. 2022 Nov 25;13:1027741. doi: 10.3389/fgene.2022.1027741 (PMC9732465; doi:10.3389/fgene.2022.1027741)

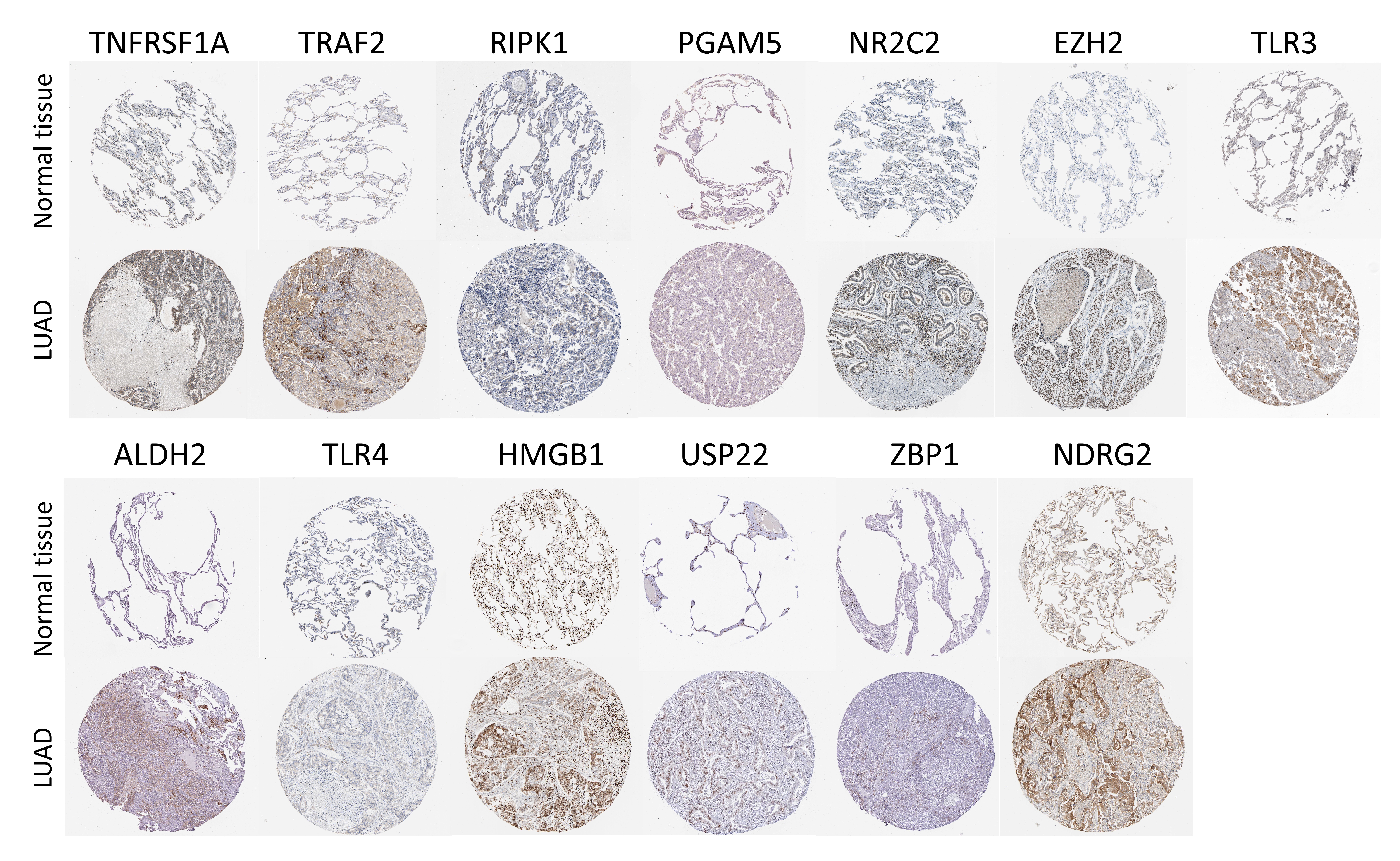

Supplement: Supplementary file 1 [file Image1.JPEG]
